# Assessment of Optimized Electrode Configuration for Electrical Impedance Myography Using Genetic Algorithm via Finite Element Model

**DOI:** 10.1155/2016/9123464

**Published:** 2016-10-24

**Authors:** Somen Baidya, Mohammad A. Ahad

**Affiliations:** Department of Electrical Engineering, Georgia Southern University, Statesboro, GA 30458, USA

## Abstract

Electrical Impedance Myography (EIM) is a noninvasive neurophysiologic technique to diagnose muscle health. Besides muscle properties, the EIM measurements vary significantly with the change of some other anatomic and nonanatomic factors such as skin fat thickness, shape and thickness of muscle, and electrode size and spacing due to its noninvasive nature of measurement. In this study, genetic algorithm was applied along with finite element model of EIM as an optimization tool in order to figure out an optimized EIM electrode setup, which is less affected by these factors, specifically muscle thickness variation, but does not compromise EIM's ability to detect muscle diseases. The results obtained suggest that a particular arrangement of electrodes and minimization of electrode surface area to its practical limit can overcome the effect of undesired factors on EIM parameters to a larger extent.

## 1. Introduction

Electrical Impedance Myography (EIM) is a four-electrode impedance measurement technique based on the fundamental principle of Ohm's law. In this technique low intensity high frequency alternating current is injected through the muscle or muscle group of interest via the outer two electrodes and the inner two electrodes record the potential difference from which the basic parameters of alternating current, resistance, reactance, and phase, are assessed [1–3]. Abnormal muscle conditions can be identified from the deviation of these parameters from normal condition [3, 4]. Because of its noninvasive nature, EIM measurement also includes the electrical response of other body tissues like subcutaneous fat thickness, muscle thickness variation [5–8], and also some nonanatomic factors like electrode surface area, interelectrode distance, and ambient temperature [9]. Studies prove that, out of different EIM parameters, reactance at a specific frequency is the desired parameter that is least affected by above-mentioned anatomic factors and is also the most effective parameter to detect muscle abnormal conditions successfully [10].

Every individual has muscle with different shape and thickness. The goal of this study is to propose* optimized* electrode configuration for this four-electrode method so that effect of muscle variation on EIM measurement is minimized. In this study, to analyse the variation of EIM parameters in different condition, a finite element model of human upper arm was developed. Finite Element Method (FEM) has been established as an appropriate approach for analysis of nonsymmetrical shape like muscle tissue for assessing alternations of muscle in disease-induced changes through ElM [11, 12]. The normal tissue properties were obtained from published resources [13] and used in this FEM model. The abnormal muscle electrical properties were obtained from sciatic crush data of rat studies [11, 14].

The optimization problem was designed to minimize the effect of different muscle thickness on desired EIM parameter, that is, reactance at 50 kHz [14] in normal condition. To simulate the optimization, the FEM model described above was incorporated in MATLAB genetic algorithm tool. Based on the properties of the FEM study, different parameters on genetic algorithm (GA) tool such as population, selection function, mutation, and crossover were set accordingly so that the problem converges to its global minima.

## 2. Methodology

The three basic parameters that are assessed easily from EIM experimentation are resistance (*R*), reactance (*X*), and phase (*θ*). The study is conducted over a large range of frequencies (typically ranges from some Hz to 1/2 MHz). These parameters show different characteristics all over the frequency domain. Deviation from the normal profile of each EIM parameters occurs due to changes in muscle electrical properties during disease progression [15]. The methodology that is implemented in EIM is expressed by Ohm's law:

(1) $$\begin{array}{c} V=IZ, \end{array}$$

where *V* is voltage, *I* is current flow, and *Z* is the impedance, which are explored in EIM for disease detection. Measured complex impedance from the experiment can be written as

(2) $$\begin{array}{c} Z=R+jX. \end{array}$$

So, the complex admittance becomes

(3) $$\begin{array}{c} Y=\frac{1}{Z}=G+jwC, \end{array}$$

where *G* is the conductance and *C* is capacitance.

Here,

(4) G=RR2+X2,C=XR2+X2w.

So the muscle's internal electrical property, conductivity, and relative permittivity depend on conductance, capacitance, and geometric factor. So the conductivity, *σ* = *K* · *G*.

And the relative permittivity, *ϵ*_*r*_ = (*K* · *C*)/*ϵ*.

**Figure 1:**
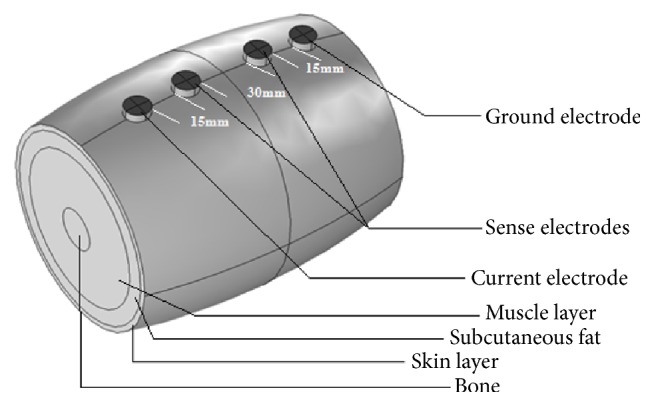
FEM model of the human upper arm using Comsol Multiphysics 4.2a (elbow to axilla) based on anatomic data. The interelectrode spacing was 15 mm-30 mm-15 mm (60 mm in total).

The FEM model of human upper arm used in this study incorporates the governing equations of EIM technique. The model was developed and analysed using the AC/DC Module, Electric Currents Physics, in Comsol Multiphysics software (Comsol, Inc., 4.2a Burlington, MA). Based on the cross-sectional view of human upper arm (Figure 1), the model was designed to have 4 different body tissue layers, that is, bone marrow, muscle, subcutaneous fat, and skin layer portrayed by four concentric cylinders with a fixed length of 14.6 cm. Thickness of the cylinders varied depending on the anatomy of the respective layer. No interelectrode capacitances or contact impedances were included [15]. The muscle and tissue material properties were homogeneous throughout the model. For nonelectrode boundaries, the normal component of the electric current was assumed to be continuous [11]. Electrodes were modelled as potential surfaces, the boundaries of which had either the excitation or zero current, except for the ground electrode, the potential of which was fixed at zero volts [15]. The discretization mesh was generated automatically with the Comsol software. At each measured frequency, longitudinal and transverse conductivities and permittivities were obtained from the rat studies and were incorporated into the model with rat data substituting for the normal human muscle. Fat, cortical bone, and marrow data were obtained over the frequency spectrum from Gabriel's dielectric survey [13]. The skin-subcutaneous fat, cortical bone, and marrow were all assumed to be isotropic but muscle is anisotropic [15]. The corresponding FEM model used for this study is illustrated in Figure 1.

As will be discussed shortly in the results, the model quite successfully follows the natural characteristics of human body to some extent. But, as mentioned above, to make EIM as an established tool for neuromuscular disease diagnosis, an optimized electrode configuration must be proposed that has least variance in accordance to other anatomic or nonanatomic factors other than muscle electrical properties. In this study, we used genetic algorithm as our optimization tool since it is one of the most effective means to find good solutions to the problems that are computationally intractable [16]. Genetic algorithm imitates the selection process found in nature by creating a random population of samples at the beginning. Then it delivers a successor population by completing a process of fitness-based choice and recombination. During recombination, first generation samples are chosen and their genetic material is recombined to produce the second generation. This then go into the next generation. In this process, a set of successive generation evolves and the average fitness of the samples tends to converge to an optimized solution [17].

The fitness function used in this study was the slope of the linear regression equation obtained from the reactance at 50 kHz as a function of muscle thickness in normal condition (discussed in detail in Appendix). The population size was set at 100, a number obtained from trial and error procedure so that the solution space is more thoroughly searched and the algorithm does not run too slow. Selection function for this specific study was chosen stochastic uniform since it samples all the solutions at evenly spaced intervals, thus minimizing the probability to pick up a local minimum rather than the global minimum. Reproduction elite count was set to 2 and crossover fraction was 0.8. The fitness function value differs slightly from the previous one with same condition due to different correlation coefficient at times. Adaptive feasible is the appropriate type of mutation in this condition. Crossover function was set as arithmetic in which the next generation populations are weighted arithmetic mean of two parents.

## 3. Results and Discussion

**Figure 2:**
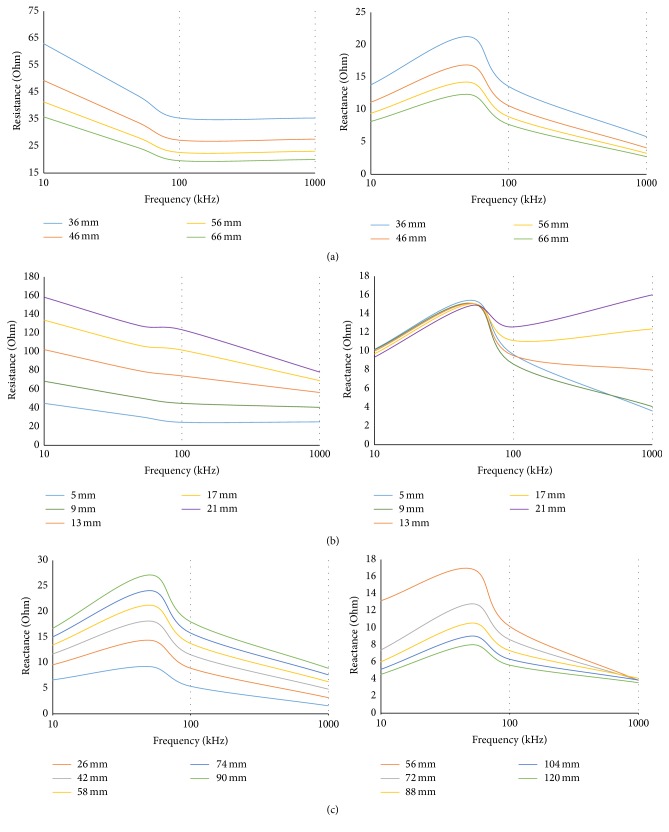
(a) Variation in EIM parameters with change in muscle thickness for (15 mm-30 mm-15 mm) electrode spacing and 65 mm × 7 mm surface electrode. (b) Variation in EIM parameters with change in skin fat thickness for (15 mm-30 mm-15 mm) electrode spacing and 65 mm × 7 mm surface electrode. (c) Variation in reactance due to different interelectrode distance between the sense electrodes (left) and the excitation electrodes (Right) for a model with 51 mm muscle thickness and 5 mm fat thickness and 65 mm × 7 mm surface electrode.

Initially, we have performed the study on varying subcutaneous fat and muscle thickness to observe the dependency of EIM parameters on these anatomic factors. As depicted in Figures 2(a) and 2(b), the percentage change of resistance in both cases is significantly larger than the percentage change of reactance for variation in fat or muscle thickness. Percentage change was determined by normalizing the desired parameter difference using change in muscle or fat thickness: that is, % change = (New  Value − Previous  value)/change  in  thickness. However, change in muscle thickness appears to have more prominent effect on percentage change of reactance than the change of subcutaneous fat. One interesting finding is, in case of large subcutaneous fat thickness, the reactance profile shows deviation from normal condition, particularly in very high frequency range. The explanation remains within the simplified circuit model of human body tissue. In case of very high frequency, both the extracellular and intracellular resistance become highly conductive. So, in case of small fat thickness the isotropic SF resistance is not that prominent as in case of larger SF thickness which also contributes to larger reactance value in high frequencies. The electrode separation used for Figure 2 was 75 mm between the excitation electrodes and 30 mm between the sense electrodes and the electrodes were 65 mm long and 7 mm in width.

Figure 2(c) illustrates how the variation in interelectrode distance affects the EIM parameters. As can be seen, placing the sense electrodes far away from each other causes the reactance profile to shoot higher at frequencies around 40 kHz.

**Figure 3:**
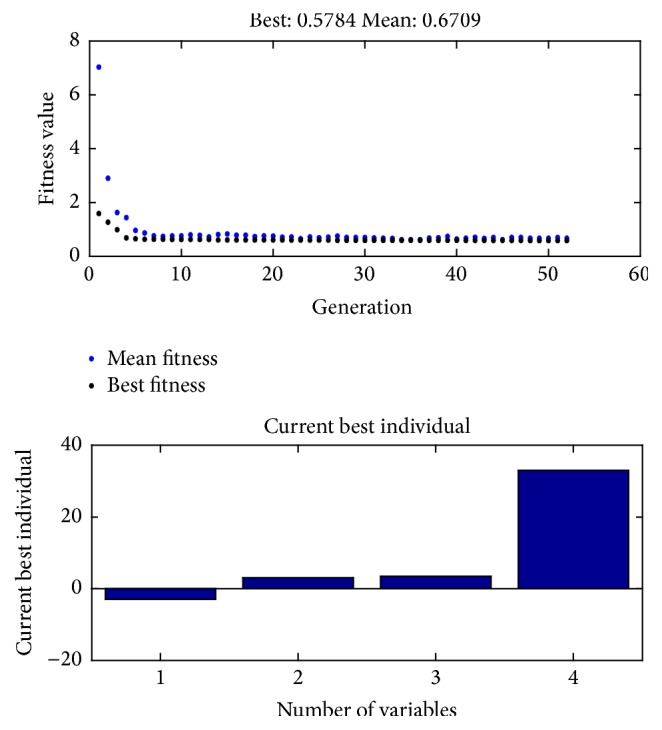
Best fitness and best individual plot from MATLAB GA tool.

The setup for varying interelectrode distance between the sense electrodes was implemented using constant 15 mm distance between the sense and excitation electrode on both sides. Separation between the excitation electrodes affects the EIM parameters in opposite manner; that is, reactance value shoots up when the excitation electrodes gets closer. That is why, we have considered the interelectrode spacing and electrode surface area as variables in our optimization problem. The goal was to minimize the alteration of EIM parameters with respect to muscle or fat thickness variation. Considering the total length of the model, the range of the interelectrode spacing was set in between 3 mm and 33 mm. The electrode surface area was designed as a variable by considering its angular coverage over the muscle model and the range was from 90 to 3 degrees on both side of the symmetry. As best fitness and best individual plots depict, the optimized electrode spacing is 87 mm between the excitation electrodes and 7 mm between the sense electrodes. The best individual score for angular coverage is 6 degrees. To simplify, the solution converges when the excitation electrodes are at their maximum limit and the sense electrodes are at their minimum limit. Since the FEM model geometry was designed using cylindrical shapes, variation in electrode surface area was incorporated as variation in angular coverage (i.e., angular coverage of 360° would refer to electrode shape that will cover the whole muscle region). Variables 1 and 2 depict the angle of coverage for which optimization occurs which were varied from 3° to 180° on both sides of the symmetry. The variation in EIM parameters also depend significantly on the area covered by the electrodes. Best individual score for surface area covered by the electrodes is also at its minimum limit. The differences between the first two variables in the best individual plot of Figure 3 are the angular coverage of the optimized electrode configuration. The third variable is the distance between sense electrodes and the fourth variable is the distance the excitation electrode should be located away from the sense electrode from optimized configuration.

**Figure 4:**
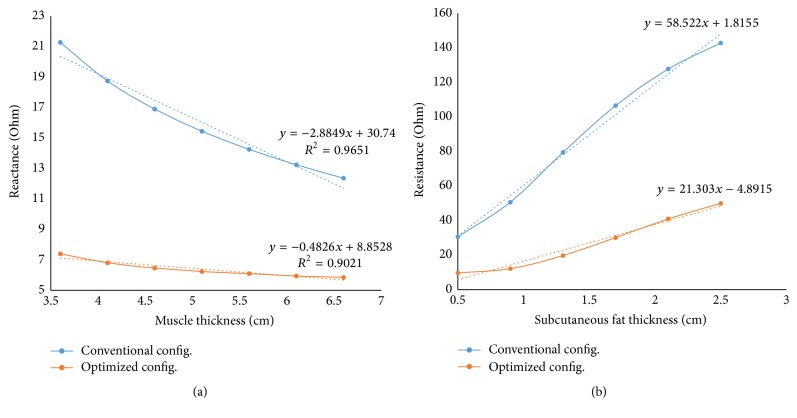
(a) Variation in reactance at 50 kHz for alteration in muscle thickness with linear regression line. (b) Variation in resistance at 50 kHz for alteration in muscle thickness with linear regression line.

Figure 4 describes the results in a more convincing way. Here, the conventional configuration is 15 mm-30 mm-15 mm spacing between the electrodes with 65 mm × 7 mm surface area. The optimized configuration is 7 mm × 7 mm surface electrodes with 33 mm-7 mm-33 mm spacing between them. Only reactance at 50 kHz has been highlighted in this figure because it has been stated in previous studies [10] that reactance at 50 kHz is the parameter which is least affected by SF thickness alteration and the best parameter to diagnose the disease effectively. As can be depicted from the figure, this parameter is less prone to change in case of optimized configuration than the conventional configuration.  Figure 4(a) depicts a reduction of 83% in the reactance variation with respect to muscle thickness for the proposed optimized configuration.  Figure 4(b) shows a 63% reduction in resistance variation with respect to fat thickness alteration. Figures 4(a) and 4(b) illustrate two different EIM parameters in two different cases because reactance is more prone to vary in case of muscle thickness variation and resistance is the parameter that is mostly affected during fat thickness change.

**Figure 5:**
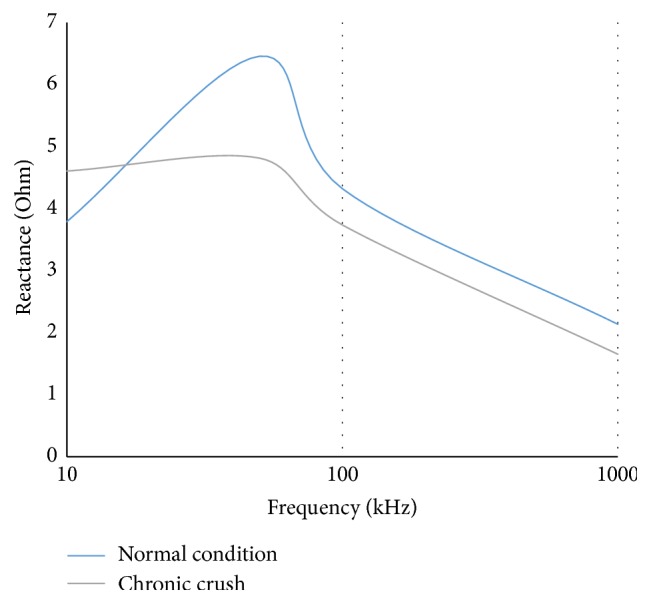
Reactance in frequency spectrum for 46 mm muscle thickness.

Besides having least dependency over other anatomic and nonanatomic factors, our major goal is to diagnose abnormal muscle condition. As can be seen from Figure 5 the proposed optimized configuration can successfully distinguish the normal muscle from the atrophied one.

**Figure 6:**
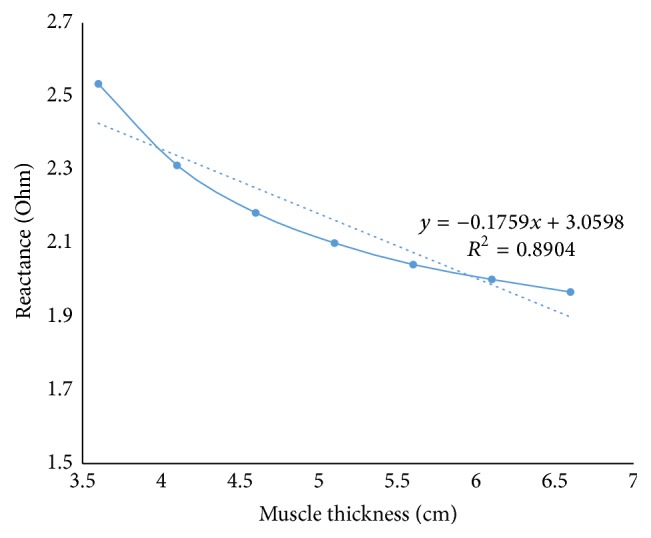
Linear regression line for 44 mm-2 mm-44 mm electrode placement with 1 mm × 1 mm surface area.

Based on the finding of the optimization problem, we carried out a more concentrated study on the electrode separation and surface area. The profile of the regression line shown in Figure 6 shows that keeping the excitation electrodes apart from each other for a larger distance than the optimized configuration and making the electrode surface area smaller make the desired EIM parameter even less variant with muscle thickness alteration. The slope of the linear regression line is 0.17, which is even smaller than the slope we got from the optimized configuration 0.48. The electrodes shape used here was 1 mm × 1 mm.

Another interesting finding of this study is the electrode shape. Based on the results of this study, it can be suggested that the less the surface area covered by the electrode the more is the stability of EIM parameters. Although the amount of injected current is the same, decreasing the surface area of electrodes results in a higher current density and larger penetration depth through body tissues especially through the subcutaneous fat thickness layer. To prove this hypothesis, we have performed another simulation with 1 mm × 1 mm electrodes placed in the same separation as the conventional electrode placement.

**Figure 7:**
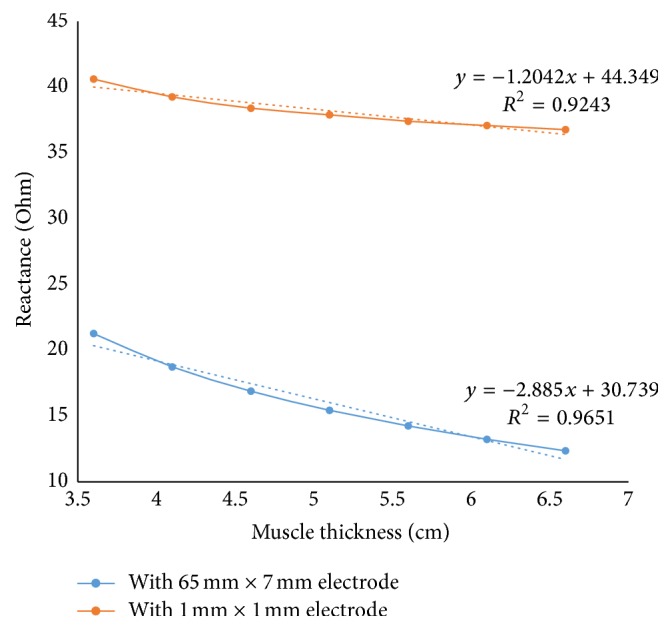
Comparison between the rectangular electrode and point electrode in 15 mm-30 mm-15 mm electrode separation for different muscle thickness.

As can be summarized from Figure 7, with the same separation between the electrodes, minimizing the electrode surface area can minimize the effect the muscle thickness alteration.

## 4. Conclusion

The objective of this study was to propose an optimized electrode configuration for which the EIM parameters are less variant with the alteration of muscle and subcutaneous fat thickness and can also detect abnormal muscle conditions. As muscle thickness increases the resistance and reactance decrease. Again, with the increment of subcutaneous fat thickness the resistance and reactance increase. And, the change in resistance due to subcutaneous fat thickness is much more prominent than the change in resistance for muscle thickness alteration and vice versa in case of reactance. So, as it seems, the resistance of the model is profoundly dominated by the isotropic property of the skin fat tissue whereas the reactance depends mostly on the anisotropic property of muscle tissue. Muscle tissue is much more conductive in nature in comparison to fat tissue. So increasing muscle thickness while keeping the skin fat layer constant provides a more conductive path to the injected current. The whole muscle fibre can be visualized as a distributed collection of infinitesimal impedance block. With larger separation between the excitation electrodes, the current is more likely to be distributed along the muscle fibre and henceforth the anisotropic property of the muscle tissue is more highlighted. Keeping the sense electrodes close enough includes a substantial part of the whole muscle membrane, thus minimizing the base value of impedance.

In practice, EIM is used as a noninvasive tool for neuromuscular disease detection and to observe the muscle condition during therapy over certain muscle group of interest. But, the anatomical diversity between subjects makes the diagnosis difficult and unreliable since there is no such EIM parameter that can distinguish different disease condition universally. The finding of this study suggests that, placing the excitation electrodes at the far end of the muscle group and the sense electrodes closer to each other up to a practical limit and more importantly, keeping the surface area of the electrodes as small as possible can eliminate the variation caused by different fat or muscle thickness for different individuals. It was also verified later in the result section that this propose setup has the ability to distinguish normal and abnormal muscle condition which drives our motivation to present this setup modification to be introduced in EIM measurement for effective implementation of the technique as a novel clinical tool. The electrode placement and shape were considered symmetrical over the whole study. From theoretical point of view, the surface area of sense electrodes was not supposed to have any impact on the EIM parameters. But, there was deviation from the expected value as we tried to keep the sense electrodes area same as conventional configuration. The scope of this study was only limited to simulation and our future plan is to extend this study by implementing it in practical experiment. It is also of concern that the optimized setup EIM parameters are considerably lower than the conventional setup value which raises the possibility of the EIM parameters being more affected by noises. This point is also subject to experimental validation.

## Appendix

The optimization problem is formulated as a single objective problem. The objective function was designed such that the effect due to anatomical alteration in muscle or fat thickness does not affect the reactance. Reactance at 50 kHz was fitted against the variation in subcutaneous fat thickness or muscle thickness variation. The variation in reactance with respect to the muscle or fat thickness change followed a linear profile. The object function was formulated to minimize the slope of the linear regression line in each electrode configuration to find the optimized electrode setup. Genetic algorithm tool was adopted in our study because of its inherent robustness and resistance to get trapped in the local minima.
